# Mitochondrial DNA drives noncanonical inflammation activation via cGAS–STING signaling pathway in retinal microvascular endothelial cells

**DOI:** 10.1186/s12964-020-00637-3

**Published:** 2020-10-28

**Authors:** Yue Guo, Ruiping Gu, Dekang Gan, Fangyuan Hu, Gang Li, Gezhi Xu

**Affiliations:** 1 Department of Ophthalmology, Eye and ENT Hospital of Fudan University, Shanghai, 200031 China; 2 Eye Institute, Eye and ENT Hospital of Fudan University, Shanghai, 200031 China; 3 Shanghai Key Laboratory of Visual Impairment and Restoration, Shanghai, 200031 China; 4 Key Laboratory of Myopia of State Health Ministry, Shanghai, 200031 China

**Keywords:** Mitochondrial DNA, Noncanonical inflammation, cGAS–STING

## Abstract

**Background:**

Pathological stimuli cause mitochondrial damage and leakage of mitochondrial DNA (mtDNA) into the cytosol, as demonstrated in many cell types. The cytosolic mtDNA then drives the activation of noninfectious inflammation. Retinal microvascular endothelial cells (RMECs) play an important role in the inner endothelial blood–retinal barrier (BRB). RMEC dysfunction frequently occurs in posterior-segment eye diseases, causing loss of vision. In this study, we investigated the involvement of cytosolic mtDNA in noninfectious immune inflammation in RMECs under pathological stimuli.

**Methods:**

RMECs were stimulated with 100 ng/ml lipopolysaccharide (LPS), 200 μM hydrogen peroxide (H_2_O_2_), or 25 mM d-glucose. After 24 h, immunofluorescent staining was used to detect the opening of the mitochondrial permeability transition pore (MPTP). Cytosolic mtDNA was detected with immunofluorescent staining and PCR after stimulation. mtDNA was then isolated and used to transfect RMECs in vitro, and the protein levels of cGAS were evaluated with western blotting. Real-time PCR was used to examine cGAS mRNA expression levels at different time points after mtDNA stimulation. The activation of STING was detected with immunofluorescent staining 6 h after mtDNA stimulation. Western blotting was used to determine the expression of STING and IFNβ, the phosphorylation status of TBK1, IRF3, and nuclear factor-κB (NF-κB) P65, and the nuclear translocation of IRF3 and NF-κB P65 at 0, 3, 6, 12, and 24 h. The mRNA expression of proinflammatory cytokines CCL4, CXCL10, and IFNB1, and transcription factor IRF1 were determined with real-time PCR, together with the concentrations of intercellular adhesion molecule 1 (ICAM-1) mRNA.

**Results:**

Pathological stimuli caused mtDNA to leak into the cytosol by opening the MPTP in RMECs after 24 h. Cytosolic mtDNA regulated the expression of cGAS and the distribution of STING in RMECs. It promoted ICAM-1, STING and IFNβ expression, TBK1, IRF3, and NF-κB phosphorylation and the nuclear translocation in RMECs at 12 and 24 h after its transfection. The mRNAs of proinflammatory cytokines CCL4, CXCL10, and IFNB1, and transcription factor IRF1 were significantly elevated at 12 and 24 h after mtDNA stimulation.

**Conclusions:**

Pathological stimulation induces mtDNA escape into the cytosol of RMECs. This cytoplasmic mtDNA is recognized by the DNA sensor cGAS, increasing the expression of inflammatory cytokines through the STING–TBK1 signaling pathway.

**Video Abstract**. (MP4 37490 kb)

**Supplementary information:**

**Supplementary information** accompanies this paper at 10.1186/s12964-020-00637-3.

## Background

The inner endothelial blood–retinal barrier (BRB) plays a crucial role in maintaining the intraocular balance and normal visual acuity. It regulates the transcellular transport between the retinal vascular and tissues, and prevents the leakage of potentially harmful agents into the retina. The breakdown of the BRB frequently occurs in posterior-segment eye diseases, causing loss of vision. BRB dysfunction is involved in many retinal diseases [1], including diabetic retinopathy, uveitis, age-related macular degeneration (AMD), retinopathy of prematurity, and retinal artery or vein occlusion. As an important part of the blood–tissue barrier, vascular endothelial cells play a critical role in posterior-segment eye diseases. Hyperglycemia, oxidative stress, inflammation, and hypoxia can injure vascular endothelial cells through a variety of complex signaling pathways [2]. Despite some breakthroughs in understanding these diseases, the common mechanism is unclear.

In recent decades, the activation of inflammation in retinal diseases has attracted increasing attention and research [3, 4]. Recent advances in understanding the role of mitochondrial damage in the inflammatory and immune responses [5] have shown that mitochondrial DNA (mtDNA) is an important source of damage-associated molecular patterns (DAMPs) [6], which trigger and maintain inflammation in some inflammatory [7] or degenerative diseases [4]. In fact, the release of mtDNA has been reported in various pathological conditions, including rheumatoid arthritis [8], cardiovascular diseases [7], trauma [9], and systemic inflammation [10]. On entering the cytoplasm, the extracellular space, or the circulation, mtDNA, acts as a DAMP, cell-type- and context-specifically engaging multiple pattern recognition receptors to trigger proinflammatory and type I interferon (IFN) responses [11]. mtDNA can activate several arms of the innate immune response, including the NLRP3 inflammasome [12], toll-like receptor 9 (TLR9) [13], and cyclic GMP-AMP synthase (cGAS)–stimulator of interferon response cGAMP interactor (STING)-driven IFN signaling [14]. In particular, mtDNA has recently been reported to be involved in the activation of cGAS, a cytosolic DNA sensor that is triggered by the escape of mtDNA into the cytosol as a consequence of mitochondrial stress [15]. In AMD is characterized by the early and induced release of mtDNA from the retinal pigment epithelium (RPE) mitochondria [4]. The escaped cytosolic mtDNA then activates the DNA sensor cGAS, which drives noncanonical inflammasome activation, resulting in RPE degeneration. In endothelial inflammation, cytosolic mtDNA activates cGAS, which induces the activation of TANK-binding kinase 1 (TBK1) and the phosphorylation of the transcription factor interferon regulatory factor 3 (IRF3), which are involved in vascular inflammation and destruction [16].

Given the importance of mtDNA–cGAS signaling in inflammation, we examined its role in retinal microvascular endothelial cells (RMECs) in this study. We found that pathological stimulation induced mitochondrial damage and the release of mtDNA in these cells, and that the cytosolic mtDNA activated the cGAS–STING pathway, which in turn induced the expression of type I IFN and proinflammatory cytokines.

## Methods

### Cell culture

Primary rat RMECs (catalogue no. RA-6065; Cell Biologics Company, Chicago, IL, USA), stored in liquid nitrogen, were quickly incubated in a 37 °C thermostatic water bath until the cell freezing medium had melted completely. The cells were then transferred to a 15 ml centrifuge tube and centrifuged at 1000×g for 5 min. The supernatant was discarded, and the cell pellet was resuspended in 10 ml of cell culture growth medium (Complete Rat Endothelial Cell Medium, catalogue no. M1266; Cell Biologics) containing 10% fetal bovine serum (FBS; lot 42F0282K, Gibco, Carlsbad, CA, USA) and 1% streptomycin–penicillin. The resuspended cells were then transferred to a T75 cell culture flask and incubated under 5% CO_2_ at 37 °C. The cell culture medium was replaced once every 2 days. The RMECs from passages 2–6 were used in the experiments. The cells were stimulated when they were > 70% confluent.

### Cell stimulations

Lipopolysaccharide (LPS; Sigma-Aldrich, St. Louis, MO, USA) was first dissolved (1 mg/ml) in phosphate-buffered saline (PBS; HyClone, Logan, UT, USA) and diluted in complete cell culture growth medium to a concentration of 100 ng/ml. Hydrogen peroxide (H_2_O_2_) solution (35%; 349,887, Sigma-Aldrich) was diluted with complete cell culture growth medium to a concentration of 200 μM. d-Glucose (WF0108, WEIAO, China) was dissolved in complete cell culture growth medium to a concentration of 25 mM. There were 2 × 10^5^ RMECs that was seeded in 6-well plates and stimulated when they were > 70% confluent. The RMECs were collected after stimulation with one or other of these treatments for 0, 12, or 24 h.

### Isolation of mitochondrial DNA and transfection

RMEC mtDNA was isolated with the Mitochondrial DNA Isolation Kit (ab65321, Abcam, Cambridge, MA, USA), according to the manufacturer’s instructions. In brief, RMECs (about 5 × 10^6^) were collected and centrifuged at 600×g for 5 min at 4 °C. The cell pellet was then washed with ice-cold PBS and centrifuged at 600×g for 5 min at 4 °C. After the supernatant was discarded, 1 × Cytosol Extraction Buffer was added to resuspend the cells and the mixture was incubated on ice for 10 min. The cells were homogenized in an ice-cold dounce tissue grinder and transferred to a 1.5 ml microcentrifuge tube. After centrifugation at 700×g for 10 min at 4 °C, the supernatant was transferred to a fresh 1.5 ml tube, and centrifuged at 10,000×g for 30 min at 4 °C. The supernatant was removed and the pellet resuspended in 1 × Cytosol Extraction Buffer and centrifuged again at 10,000×g for 30 min at 4 °C. Enzyme Mix (5 μl) was added to the tube, which was incubated in a 50 °C water bath for 60 min before centrifugation in a microcentrifuge at the top speed for 5 min at room temperature. The pellet was mtDNA. After the supernatant was removed, Tris–EDTA (TE) buffer was used to resuspend the mtDNA. The concentration of mtDNA was measured with a NanoDrop 2000 spectrophotometer (Thermo Fisher Scientific, Rockford, IL, USA).

RMECs were transfected with the mtDNA using Attractene Transfection Reagent (Qiagen, 301,005), according to the manufacturer’s instructions. Briefly, the cells were incubated under normal growth conditions (37 °C and 5% CO_2_) and were 70% confluent on the day of transfection. The mtDNA was diluted in TE buffer to a concentration of 0.5 μg/μl and then 2 μl of mtDNA was mixed with growth medium containing no serum or antibiotics to a total volume of 100 μl. Attractene Transfection Reagent (4.5 μl) was added to the Eppendorf tube and the transfection complexes were allowed to form. After the solution was pipetted up and down or vortexed, the tube was centrifuged for a few seconds to make the transfection complexes be at the bottom of the tube. The samples were incubated for 15 min at room temperature to allow the transfection complex formation. The medium was replaced with new complete cell culture medium, and the mixture of mtDNA and Attractene Transfection Reagent was added dropwise onto the cells. The plate was gently swirled to ensure the uniform distribution of the transfection complexes. The cells were incubated under their normal growth conditions and the medium was replaced again after 3 h.

### Detection of mitochondrial permeability transition pore opening

The opening of the mitochondrial permeability transition pore (MPTP) was detected with the Image-iT™ LIVE Mitochondrial Transition Pore Assay Kit (Invitrogen, I35103), according to the manufacturer’s instructions. RMECs were cultured in a 24-well plate in complete cell growth medium in the presence of LPS (1 mg/mL), H_2_O_2_ (200 μM), or high glucose (25 mM) for 24 h. The cells were washed in 1 × Hank’s balanced salt solution (HBSS; Corning). Calcein AM stock solution (1.0 mM), MitoTracker Red CMXRos stock solution (200 μM), and 1.0 mM Hoechst 33342 dye were then combined (1.0 μL of each). Prewarmed (37 °C) HBSS (997 μl) was added to the preparation to produce the labeling solution. A sufficient amount of labeling solution was applied to cover cells adhering to a coverslip. The cells were incubated for 15 min at 37 °C in the dark. A mixture of 1.0 μl of 1.0 M CoCl_2_ and 999 μl of HBSS was then added and the cells were incubated for another 15 min at 37 °C in the dark. The cells were washed twice with warm HBSS buffer to remove any residual dye and to minimize the background noise, and mounted in warm buffer. The cells were observed under a Leica TCS SP8 WLL confocal microscope with a 63×, 1.35-NA oil-immersion objective. The approximate excitation/emission peaks of calcein after hydrolysis were 494/517 nm, respectively; the approximate excitation/emission peaks of MitoTracker Red CMXRos dye were 579/599 nm, respectively; and the approximate excitation/emission peaks of Hoechst 33342 dye were 350/461 nm, respectively. The calcein, MitoTracker Red CMXRos, and Hoechst 33342 signals were observed with standard filter sets.

### Detection of mtDNA and cytosolic DNA with staining

Immunofluorescent double-label staining was used to assess the release of mtDNA into the cytoplasm. After 24 h in the presence of LPS, H_2_O_2_, or high glucose, RMECs were washed twice with PBS. MitoTracker Red stock solution (1 mM; MitoTracker™ Red CMXRos - Special Packaging, Invitrogen, M7512) was diluted in growth medium without FBS. The prewarmed (37 °C) staining solution containing MitoTracker Red was added to the cells in a 24-well plate to a final working concentration of 400 nM. The cells were cultured for 15–45 min under standard growth conditions in the dark. Quant-iT™ PicoGreen™ dsDNA Reagent (Invitrogen, P7581) was diluted 200-fold, according the manufacturer’s instructions. After MitoTracker Red staining solution was removed and the cells were washed twice with PBS. An aliquot (1.0 ml) of the aqueous working solution of Quant-iT™ PicoGreen dsDNA Reagent was added to each cell sample, which was incubated for 10 min at 37 °C in the dark. The PicoGreen reagent was discarded and the cells were rinsed with 4′,6-diamidino-2-phenylindole (DAPI; Sigma-Aldrich) solution for 10 min. After staining was complete, the staining solution was replaced with fresh prewarmed medium or buffer, and the cells were observed with a fluorescence confocal microscope (Leica TCS SP8 WLL). The approximate excitation/emission peaks of MitoTracker Red were 579/599 nm, respectively, and the approximate excitation/emission peaks of Quant-iT™ PicoGreen dsDNA Reagent were 502/523 nm, respectively.

### Detection of cytosolic mtDNA with PCR

To detect cytosolic mtDNA with a PCR assay, the cytosol of RMECs was extracted with a Mitochondria Isolation Kit for Cultured Cells (C3601, Beyotime, China), according to the manufacturer’s instructions. Ice-cold PBS was used to wash the RMECs, which were then centrifuged at 600×g for 5 min at 4 °C. The supernatant was removed and discarded. The pellet was resuspended in Mitochondria Isolation Reagent and incubated for 15 min on ice. The cell suspension was transferred to a dounce tissue grinder and homogenized with 15 passes on ice. The homogenate was transferred to a 1.5 ml microcentrifuge tube, and centrifuged at 600×g for 10 min at 4 °C. The supernatant was transferred to a fresh 1.5 ml tube and centrifuged at 11,000×g for 10 min at 4 °C. The cytosolic supernatant was collected. The FlexiGene DNA Kit (no. 51206, Qiagen) was used to isolate the mtDNA from the collected cytoplasm. Buffer FG1 was added to the collected cytoplasm in a 2 ml Eppendorf tube and mixed by pipetting. Buffer FG2 was then added and the tube was inverted three times. The tube was placed in a heating block and incubated at 65 °C for 10 min. Isopropanol (100%) was added and the tube was inverted to induce DNA precipitation. After centrifugation for 3 min at 10,000×g, the supernatant was discarded and Buffer FG3 was added to dissolve the DNA during incubation for 30 min at 65 °C in a heating block. The mtDNA was detected with quantitative PCR with primers that hybridized to sequences in the gene encoding mitochondrial cytochrome c oxidase 1 *(mt-Co1)*. The nuclear DNA was measured with PCR using primers that hybridized sequences in the 18S rDNA (encoding 18S rRNA). The copy numbers of mtDNA were normalized against the copy numbers of nuclear DNA, and compared between groups. All PCR primers were synthesized by Tsingke Biological Technology (Shanghai, China). The primers for 18S rDNA were 5′-TAGAGGGACAAGTGGCGTTC-3′ (forward) and 5′-CGCTGAGCCAGTCAGTGT-3′ (reverse). The primers for *mt-Co1* were 5′-GCCCCCGATATGGCGTTT-3′ (forward) and 5′-GTTCAACCTGTTCCTGCTCC-3′ (reverse).

### Western blotting analysis

Each sample was collected and suspended in lysis buffer (Cell Signaling Technology, Beverly, MA, USA). The mixture was centrifuged at 10,000×g for 15 min. The BCA Protein Assay Kit (Beyotime, Shanghai, China) was used to determine the protein concentrations. Cytoplasmic Extraction Reagents (Thermo Fisher Scientific) were used to separate the nuclear proteins and cytoplasmic proteins. The proteins were separated with SDS-PAGE and transferred to polyvinylidene difluoride membranes (Millipore, Billerica, MA, USA). The membranes were blocked with 5% bovine serum albumin for 1 h and incubated with the primary antibody overnight. The antibodies used for this assay were: anti-cGAS antibody (ab179785, Abcam), anti-ICAM1 [1A29] antibody (ab171123, Abcam), anti-TBK1/NAK (D1B4) rabbit mAb (#3504, Cell Signaling Technology), anti-phospho-TBK1/NAK (Ser172) (D52C2) XP® rabbit mAb (#5483, Cell Signaling Technology), anti-IRF3 (D83B9) rabbit mAb (#4302, Cell Signaling Technology), anti-phospho-IRF3 (Ser396) (D6O1M) rabbit mAb (#29047, Cell Signaling Technology), anti-NF-κB P65 (D14E12) XP rabbit mAb (#8242, Cell Signaling Technology), anti-phospho-NF-κB P65 (Ser536) (93H1) rabbit mAb (#3033, Cell Signaling Technology), anti-STING (D1V5L) Rabbit mAb (#50494, Cell Signaling Technology), anti-Interferon beta antibody (ab140211,abcam), anti-β actin antibody (ab8227, Abcam), anti-lamin B1 antibody (a nuclear envelope marker; ab16048, Abcam), and horseradish-peroxidase-conjugated goat anti-rabbit IgG H&L (ab205718, Abcam). After the membranes were washed three times with 1 × TBS–Tween buffer, they were incubated with the secondary antibody for 1 h at room temperature. Pierce ECL Western Blotting Substrate (Thermo Fisher Scientific) and a Kodak Digital Imaging System (Kodak, Rochester, NY, USA) were used to visualize and quantify the immunoblots.

### Immunofluorescence

Coverslips were placed in 24-well plates before they were seeded with 5 × 10^4^ cells. After stimulation, the RMECs were washed twice with PBS and fixed with 4% paraformaldehyde for 10 min. The cells were washed again and the cultures incubated with blocking buffer containing 0.3% Triton X-100 and 5% goat serum for 1 h at 37 °C. They were then incubated overnight at 4 °C with the primary antibody (anti-TMEM173/STING antibody, diluted 1:50; cat 19,851–1-AP, Proteintech) and co-stained with ER, ERGIC and Golgi maker respectively (Calnexin Monoclonal Antibody (GT1563), diluted 1:100; cat MA5–31501, Invitrogen, LMAN1 Monoclonal Antibody (OTI1A8), diluted 1:100; cat MA5–25345, Invitrogen, Purified Mouse Anti-GM130, diluted 1:100; cat 610,822, BD bioscience). After the RMECs were rinsed with PBS, they were incubated with the secondary antibody (Alexa-Fluor®-488-conjugated goat anti-rabbit IgG H&L, diluted 1:1000; ab150077, Abcam, Alexa-Fluor®-555-conjugated goat anti-mouse IgG H&L, diluted 1:1000; ab150114, Abcam) for 60 min at room temperature and counterstained with DAPI (Sigma-Aldrich) for another 10 min. The cells were washed twice with PBS and observed with a laser confocal microscope (Leica Microsystems).

### Real-time PCR

RMECs were treated as described above and the total RNA was isolated with TRIzol Reagent (Invitrogen). The mRNA was reverse transcribed into cDNA with the PrimeScript RT reagent Kit (Takara, Shiga, Japan), according to the manufacturer’s protocol. The LightCycler 480 II real-time PCR instrument (Roche, Basel, Switzerland) was used to perform real-time PCR in 10 μl reaction mixtures containing 0.2 μl of the forward primer, 0.2 μl of the reverse primer, 1 μl of cDNA, 5 μl of 23 LightCycler 480 SYBR Green I Master Mix (Roche), and 3. 6 μl of nuclease-free water. The thermal cycling parameters were 95 °C for 10 min, followed by 40 cycles of 95 °C for 10 s and 60 °C for 30 s. The mRNA levels were normalized to those of *ACTB* mRNA and calculated with the 2^−ΔΔCt^ method. All PCR primers were synthesized by Tsingke Biological Technology (Shanghai, China). The primers are shown in Table S1.

### Statistical analysis

All statistical analyses were performed with SPSS for Windows version 17.0 (SPSS, Inc., Chicago, IL, USA). Data are presented as means ± standard deviations. Representative images of all assays are shown, and the quantitative results are the means of three independent experiments. Differences between two groups were compared with the Mann–Whitney *U* test. To compare differences among three or more groups, the Kruskal–Wallis test was used in this study. Values of *P* < 0.05 were considered statistically significant.

## Results

### Pathological stimuli cause mtDNA to leak into the cytosol by opening the MPTP in RMECs

**Fig. 1:**
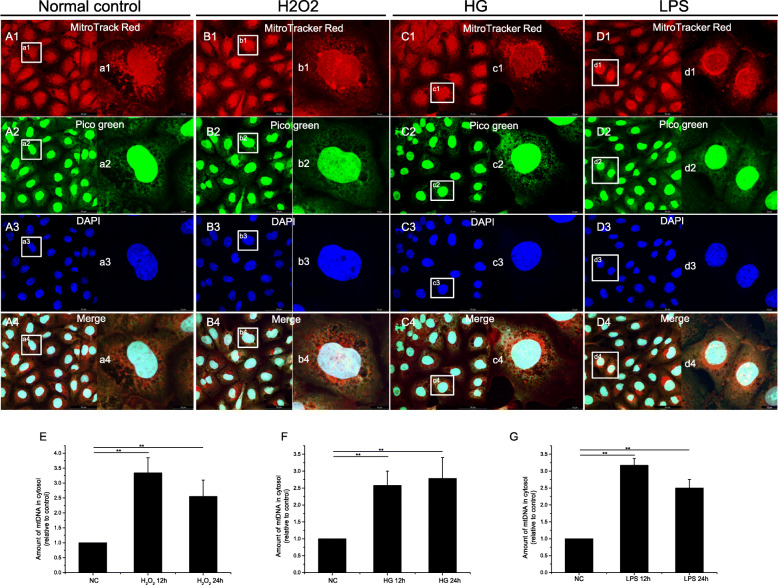
(1) Immunofluorescent double-labeling of DNA and mitochondria in RMECs after treatment with 200 μM H2O2 (B1–4), 25 mM glucose (C1–4), or 100 ng/ml LPS (D1–4) for 24 h. (A1, B1, C1, and D1) Mitochondria were labeled with MitoTracker Red. (A2, B2, C2, and D2) Double-stranded DNA was labeled with PicoGreen. (A3, B3, C3, and D3) Nuclei were labeled with DAPI. (A4, B4, C4, and D4) Merged images of A1–3, B1–3, C1–3, and D1–3, respectively. (a1–4, b1–4, c1–4, and d1–4) Magnification of A1–4, B1–4, C1–4, and D1–4, respectively. (2) Real-time PCR was used to determine the copy number of mtDNA in the cytoplasm. In RMECs, the copies of mtDNA released from the mitochondria are shown after treatment with 200 μM H2O2 (E), 25 mM glucose (F), or 100 ng/ml LPS (G) for 0, 12, and 24 h. ∗∗*P* < 0.01, *n* = 3 biological repeats in each group

We used double-stranded DNA (dsDNA) staining (PicoGreen) and mitochondrial staining (MitoTracker) to simultaneously detect mtDNA (PicoGreen that colocalized with MitoTracker), nuclear DNA (PicoGreen in the nucleus), and cytosolic DNA (PicoGreen that did not colocalize with either MitoTracker or the nucleus). As shown in Fig. 1a-d, small dsDNA particles were detected outside the mitochondria and the nuclei in the RMECs treated with LPS, H_2_O_2_, or high glucose, suggesting the presence of cytosolic DNA. However, cytosolic DNA was barely detectable in the untreated RMECs. We also detected mtDNA in the cytosolic fractions with a PCR analysis using primers directed against mtDNA sequences. As shown in Fig. 1e-g, the amount of cytosolic mtDNA was significantly higher in cells treated with H2O2, high glucose, or LPS than in the control cells. Together, these data suggest that pathological stimuli cause mtDNA leakage into the cytosol.

**Fig. 2:**
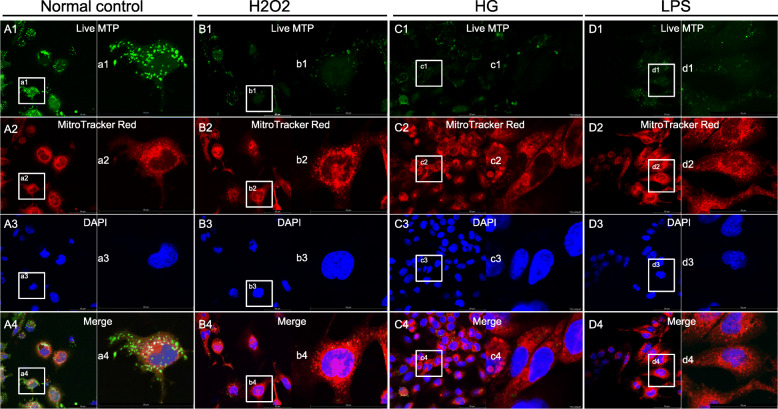
Mitochondrial permeability transition pore (MPTP) opening was detected with Image-iT™ LIVE Mitochondrial Transition Pore Assay Kit. (A1, B1, C1, and D1) show the fluorescence of calcein (green). (A2, B2, C2, and D2) show MitoTracker Red staining. (A3, B3, C3, and D3) show DAPI staining. (A4, B4, C4, and D4) Merged images of A1–3, B1–3, C1–3, and D1–3, respectively. (a1–4, b1–4, c1–4, and d1–4) Magnification of A1–4, B1–4, C1–4, and D1–4, respectively

How is mtDNA released into the cytoplasm? Previous research ascribed the escape of mtDNA to the opening of the MPTP after pathological stimulation. Therefore, we examined the function of the MPTPs in RMECs at 24 h after H2O2, high-glucose, or LPS treatment. The opening of the MPTP was detected with the Image-iT™ LIVE Mitochondrial Transition Pore Assay Kit after stimulation, but was not observed in the unstimulated cells (Fig. 2).

### Cytosolic mtDNA regulates the expression of cGAS and the distribution of STING in RMECs

**Fig. 3:**
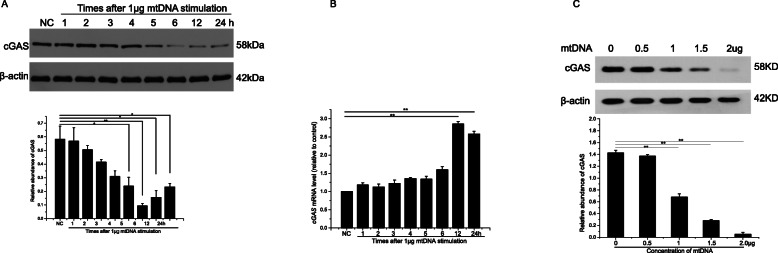
Changes in cGAS protein and mRNA levels in RMECs after mtDNA stimulation. **a** RMECs were treated with 1 μg of mtDNA for 1 h, 2 h, 3 h, 4 h, 5 h, 6 h, 12 h and 24 h. And the cGAS protein levels were determined by Western blot analysis; β-actin was used as the loading control. Quantitative analysis of cGAS, as determined by densitometric analysis, expressed as a ratio of β-actin. **b** RT–PCR was used to determine *cGAS* mRNA levels in RMECs treated with 1 μg of mtDNA for different times. **c** Western blotting analysis of cGAS protein levels in RMECs treated with different concentrations of mtDNA for 6 h. ∗*P* < 0.05, ∗∗*P* < 0.01, *n* = 3 in each group

cGAS, an important cytosolic DNA sensor, decreased soon after mtDNA transfection and was lowest 6 h after stimulation. The level of cGAS protein then increased gradually to 12 and 24 h after stimulation (Fig. 3a). This increase was consistent with the changes in *cGAS* mRNA. As shown in Fig. 3b, *cGAS* mRNA remained stable for 5 h after mtDNA stimulation, increased slightly at 6 h, and increased significantly at 12 and 24 h. The decline in cGAS within the first 6 h correlated positively with the concentration of transfected mtDNA (Fig. 3c).

**Fig. 4:**
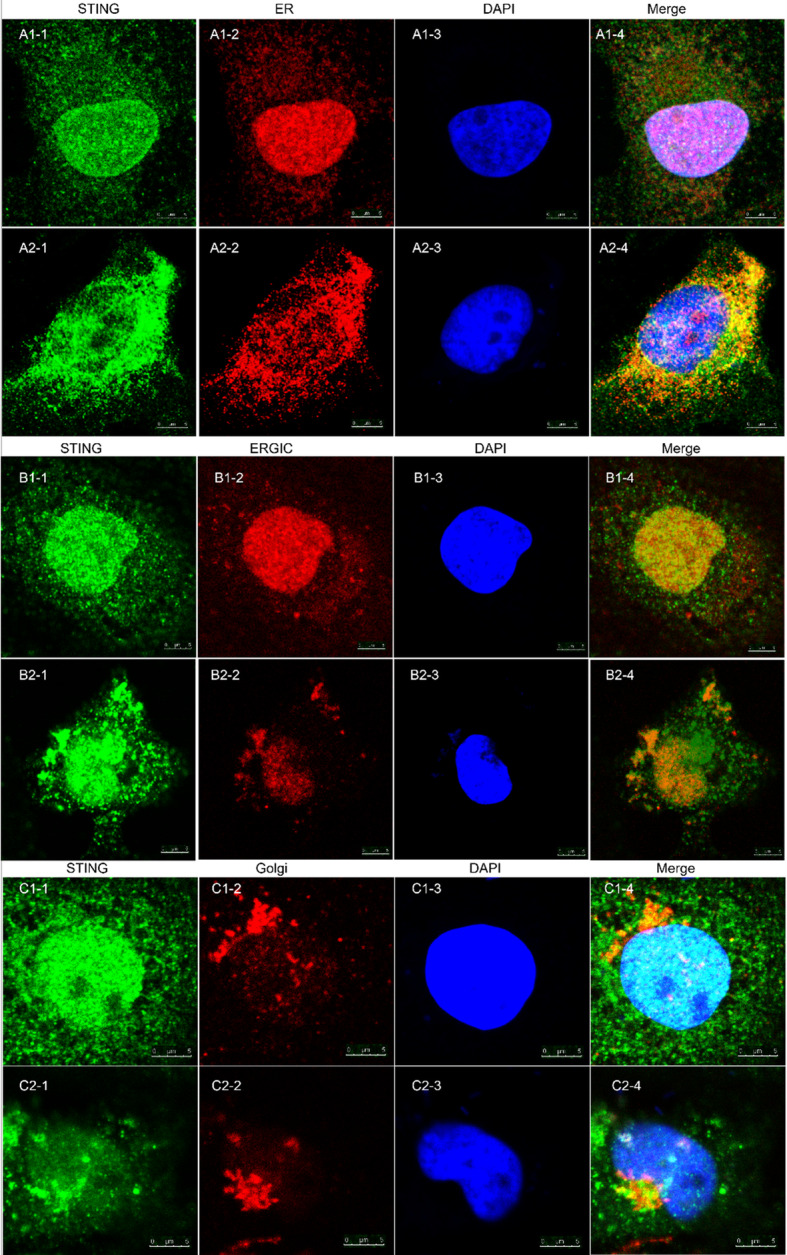
Immunofluorescence of ER, ERGIC and Golgi and STING after mtDNA stimulation of RMECs. Figure A1-C1: normal control groups; Figure A2-C2: mtDNA stimulation groups. Green was STING and blue was DAPI. Red was ER, ERGIC and Golgi in figure A2–2, B2–2 and C2–2, respectively. In normal control group, STING was dispersive and co-localization with ER maker. In mtDNA-stimulated group, STING was aggregated specks formed in the perinuclear region and partly co-localization in ERGIC and Golgi makers

STING, an endoplasmic-reticulum (ER)-membrane protein, is a critical signaling molecule of the immune system and is activated by the leakage of mtDNA into the cytosol. At normal control group of RMECs, STING was dispersive and localization in ER in cytoplasm. Then the pronounced translocation of STING was observed, with the formation of aggregated specks in the perinuclear region at 6 h after stimulation with 1.0 μg of mtDNA. Furthermore, the STING moved from ER and partly localized in ERGIC and Golgi in cytoplasm after mtDNA stimulation (Fig. 4).

### Cytosolic mtDNA promotes ICAM-1, STING and IFNβ expression; TBK1, IRF3, and NF-κB phosphorylation; IRF3, and p65 NF-κB nuclear translocation in RMECs

**Fig. 5:**
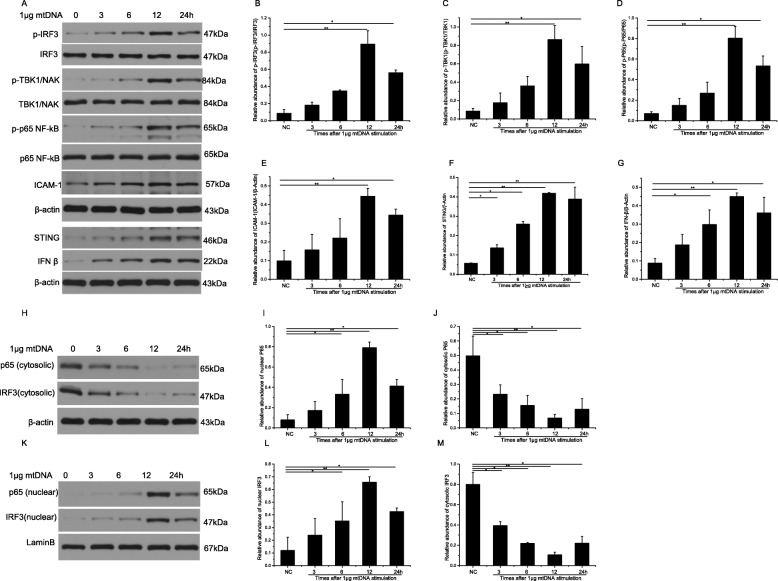
Western blotting analysis of phospho-IRF3 (**b**), TBK1 (**c**), and NF-κB P65 (**d**), ICAM1 (**e**), STING (**f**) and INF-β (**g**) in RMECs after stimulation with 1 μg of mtDNA for different times. Western blotting analysis of IRF3 and NF-κB P65 levels in the cytoplasm and nuclei of RMECs after stimulation with 1 μg of mtDNA for different times. **h, j, m** Amounts of NF-κB P65 and IRF3 in the cytoplasm. **k, i, l** Amounts of NF-κB P65 and IRF3 in the nuclei. ∗∗*P* < 0.01, *n* = 3 biological repeats in each group

As shown in Fig. 5a-d, the phosphorylation of IRF3, TBK1, and nuclear factor-κB (NF-κB) P65 was significantly increased by mtDNA at 12 and 24 h after transfection. The mtDNA stimulation also promoted protein levels of ICAM-1at 12 and 24 h, STING at 3, 6, 12 and 24 h and IFNβ at 6, 12 and 24 h Fig. 5e-g. Western blotting analysis of IRF3 and p65 levels in the cytoplasm and nuclei of RMECs after stimulation with 1 μg of mtDNA for different times. Figure 5h-m shown that most IRF3 and p65 were located in the cytosol, with little nuclear p65 in normal RMECs. The stimulation of mtDNA significantly reduced the levels of cytosolic IRF3 and p65 at 3, 6,12 and 24 h and induced marked increase in nuclear IRF3 and p65 at 6,12 and 24 h. In a word, stimulation of mtDNA significantly enhanced p65 translocation from the cytoplasm to the nucleus.

### Cytosolic mtDNA promotes transcription of proinflammatory cytokines in RMECs

**Fig. 6:**
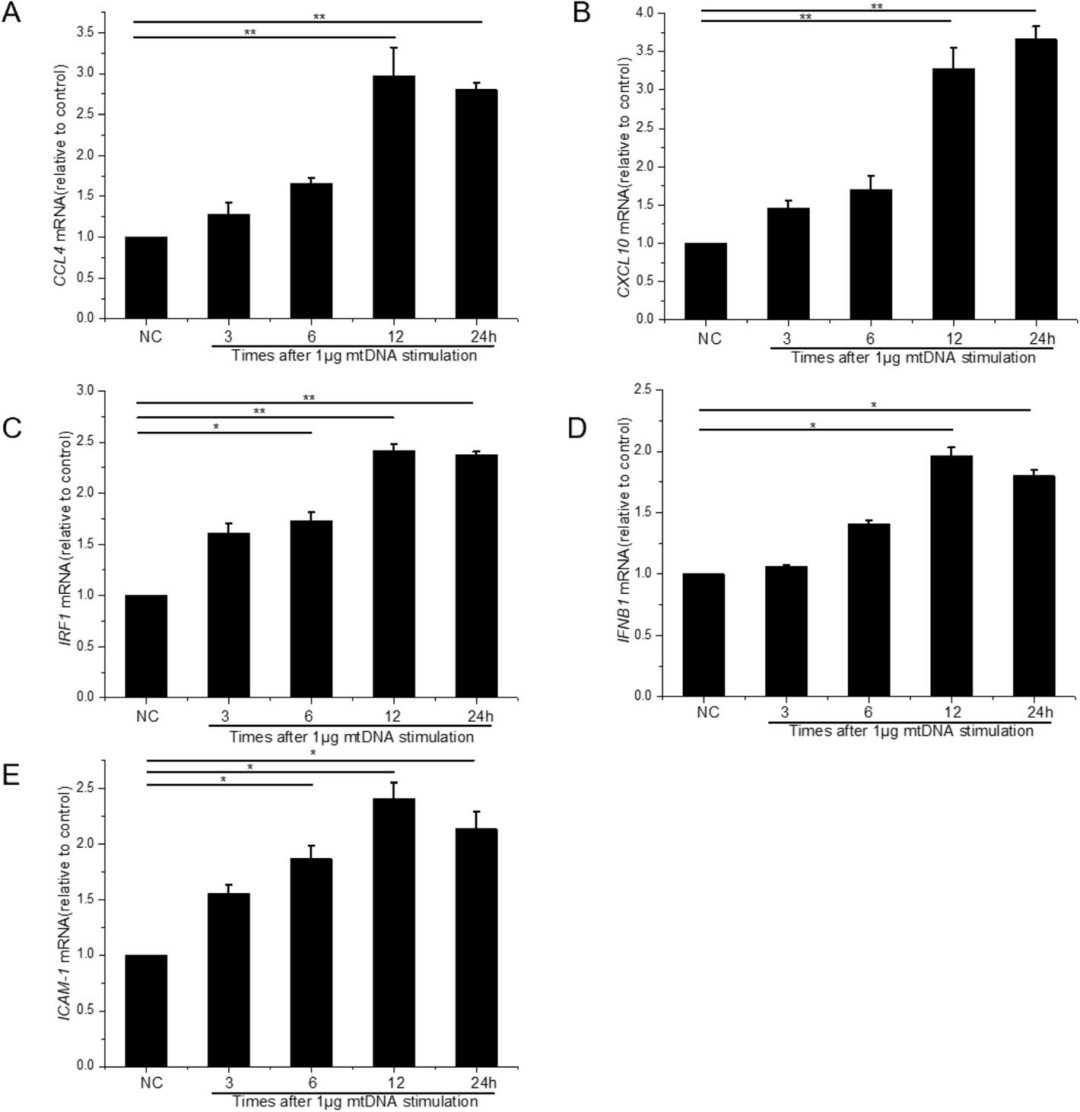
Real-time PCR was used to evaluate the transcription of *CCL4* (**a**), *CXCL10* (**b**), *IRF1* (**c**), *IFNB1* (**d**), and *ICAM1* (**e**) in RMECs after stimulation with 1 μg of mtDNA for different times. ∗*P* < 0.05, ∗∗*P* < 0.01, *n* = 3 biological repeats in each group

In parallel with the nuclear translocation of IRF3 and NF-κB P65, the amount of intercellular adhesion molecule 1 (ICAM1) increased at both the protein and mRNA levels after mtDNA stimulation (Figs. 5e and 6e). Simultaneously, cytosolic mtDNA significantly induced the transcription of *CCL4*, *CXCL10*, *IRF1*, and *IFNB1*. As shown in Fig. 6, *CCL4*, *CXCL10*, and *IFNB1* mRNAs were significantly higher at 12 and 24 h than in the normal control. *IRF1* and *ICAM1* mRNAs increased at 6 h and continued to increase to 24 h.

## Discussion

In this study, we have shown that the escape of mtDNA into the cytosol under pathological stimulation is a universal phenomenon in RMECs. The release of mtDNA from the mitochondria may occur after the opening of the MPTP. The cytoplasmic mtDNA is recognized by the DNA sensor cGAS in the cytosol, which is followed by the activation of STING. Activated STING translocates to the perinuclear region, where it forms aggregated specks, and then promotes the phosphorylation of TBK1, IRF3, and NF-κB P65 and the nuclear translocation of IRF3 and NF-κB P65. With the nuclear transfer of IRF3 and NF-κB P65, the expression of downstream factors (e.g. ICAM1, CCL4, CXCL10, IRF1, and IFN-β1) is induced after the extracted mtDNA is transfected into RMECs.

The mitochondria play an important role not only in the generation of cellular energy but also in the metabolism of lipids and amino acids [17]. The complex structure and highly dynamic nature of the mitochondria are thought to make them vulnerable to pathological conditions [18]. Blajszczak noted [19] that mtDNA is more often damaged than nuclear DNA because of its specialized location in the inner mitochondrial membrane. Previous reports have suggested that mitochondria evolved from α-Proteobacteria and that the structure of the eukaryotic mtDNA contains hypomethylated CpG motifs that have some similarity to bacterial CpG DNA [20, 21]. Several recent studies have demonstrated that mitochondria are versatile organelles that function in infection, inflammation, cancer, and degenerative diseases [22]. Nerlich reported that under pathological stimulation, the Ca^2+^ influx into the mitochondria changes [23], accompanied by morphological changes in the mitochondria [24]. The mitochondrial Ca^2+^ influx and loss of mitochondrial membrane potential results in the opening of the MPTP, with the consequent release of cytochrome c and mtDNA into cytoplasm [23, 25]. In this study, we have shown that pathogenic stimulation causes MPTP opening and the escape of mtDNA, which was detected with immunofluorescence. This activated a noninfectious inflammatory response in RMECs through the cGAS–STING pathway. Moreover, this is a general phenomenon in RMECs when mtDNA escapes from the mitochondria.

The structure of cGAS contains a nucleotidyltransferase domain and two major DNA-binding domains, which recognize dsDNA, combine ATP and GTP, and synthesize cyclic GMP-AMP (cGAMP) [26]. It has been recognized as an important cellular DNA sensor in recent years [27]. As a cytosolic DNA sensor, the cGAS played as a conserved enzyme and converted ATP and GTP into the dinucleotide cGAMP. The cGAMP further played as a second messenger and activated downstream cGAMP-STING signaling pathway. Then the cGAS was degraded by p62-depdendent ubiquitination as previous research reported [28]. In our present research, the amount of cGAS decreased at 1 h and was lowest at 6 h after mtDNA stimulation. At 12 and 24 h after mtDNA stimulation, the transcription and translation of cGAS tended to increase gradually. The STING plays an important role in facilitating the innate immune signaling processes during DNA virus infection [29]. In normal RMECs, the STING was dispersive and localization in cytoplasm and co-localized in ER. After 1μg mtDNA stimulation, the pronounced translocation of STING was observed, with the formation of aggregated specks in the perinuclear region and partly co-localization in ERGIC and Golgi. The changes indicated that STING was activated and moved from ER to ERGIC and Golgi. Research has shown that activated STING enhances the phosphorylation of protein kinases IκB kinase (IKK) and TANK-binding kinase 1 (TBK1) [30, 31]. This then induces the phosphorylation and nuclear translocation of the transcription factor IRF3 [32]. Our data are consistent with these studies. After treatment with mtDNA, the phosphorylation of TBK1 and IRF3 gradually increased and IRF3 aggregated in the nucleus rather than in the cytosol. NF-κB is a regulatory molecule that promotes the expression of some proinflammatory factors, and is also phosphorylated as a consequence of the full activation of TBK1 [33]. In this study, we confirmed that mtDNA resulted in NF-κB phosphorylation and nuclear transfer via the cGAS–STING pathway. In another study, Mao et al. demonstrated that activated IRF3 is an essential factor mediating *ICAM1* expression, and that IRF3 binds directly to the *ICAM1* promoter and induces *ICAM1* expression during inflammation [16]. In a study of diabetic retinopathy induced by hyperglycemia, the transcript and protein levels of ICAM1 were upregulated [34]. ICAM1 has already been identified as playing a key role in diabetic retinopathy and vascular inflammation, and the knockdown of ICAM1 can reduce endothelial injury and the loss of pericytes [34]. In this study, we found that the production of inflammatory cytokines, such as CCL4, CXCL10, IRF1, and IFN-β1, was accompanied by the phosphorylation of IRF3 and NF-κB. Macrophage inflammatory protein 1β (MIP1β/CCL4) is a highly related member of the CC chemokine subfamily [35] that is involved in the occurrence and development of atherosclerosis [36], and CXCL10 (IP-10) is a chemokine induced by IFN-γ. It is chemoattractive to immune cells and enhances angiogenesis in cancers and autoimmune diseases [37]. Several studies by Shizuka et al. reported that increases in MIP1β and IP-10 occur in the vitreous fluid during rhegmatogenous retinal detachment, proliferative diabetic retinopathy, and retinal vein occlusion [38, 39]. IRF3 is responsible for the induction of some IFN-stimulated genes [40]. Type I interferon (*IFNB1*) expression is upregulated by the phosphorylation of STAT6, which occurs after the phosphorylation of IRF3 in the STING signaling pathway [41]. A study by Wang et al. suggested that IFN-β1 protein is significantly increased via the TLR4 Inflammatory pathway during the breakdown of the BRB in diabetic retinopathy [42]. IFN-β1 also contributes to the initiation of the innate immune response by binding the TLRs on RPE cells. *IRF1* is a known target gene of NF-κB, which binds directly to its promoter, stimulating the expression of several genes related to the host defense against pathogens [43]. A previous study suggested that IRF1 contributes to the activation of complement factor H, a key regulator of AMD [44]. A study by Eckhardt et al. demonstrated that IRF1 is a critical regulator of the proinflammatory cytokine secretion induced by BV6 during the immune response [45]. Stimulation with LPS induced mtDNA replication and the activation of IRF1 and NLRP3, which is similar to our findings in cells treated with isolated mtDNA [12]. In our study, pathological stimulation led to the release of mtDNA into cytoplasm of RMECs, which induced a noninfectious immunoinflammatory response. This was accompanied by the secretion of a series of inflammatory factors, which mediate the adhesion of inflammatory cells around vascular endothelial cells. The BRB function was destroyed as a consequence of this signaling cascade in the vascular inflammatory response.

## Conclusion

The cGAS–STING signaling pathway is a conserved pathway that plays a critical role in the immune and inflammatory responses [46]. Although there have been several reports of the involvement of cGAS–cGAMP–STING signaling in cancer, degenerative diseases, and systemic inflammation, there have been few studies of diseases related to the inner BRB in the retina [4, 22]. We have demonstrated that pathological stimulation induces mtDNA escape into the cytosol of RMECs, and that this cytoplasmic mtDNA is recognized by the DNA senor cGAS, increasing the expression of inflammatory cytokines through the STING–TBK1 signaling pathway. Our findings suggest that mtDNA escape is one of the most important factors in the destruction of the BRB in retinal vascular diseases, such as diabetic retinopathy, uveitis, and AMD.

## Supplementary information

**Additional file 1: Table S1.** Primer sequences used for real-time PCR in this study. (DOCX 15 kb)

**Additional file 2.** (TIFF 458 kb)

## Data Availability

Not applicable.

## Abbreviations

mtDNA: Mitochondrial DNA
RMECs: Retinal microvascular endothelial cells
BRB: Blood–retinal barrier
LPS: Lipopolysaccharide
H2O2: Hydrogen peroxide
HG: High Glucose
MPTP: Mitochondrial permeability transition pore
cGAS: Cyclic GMP-AMP synthase
TBK1: TANK-binding kinase 1
IRF3: Interferon regulatory factor 3
NF-κB: Nuclear factor-κB
CCL4: Macrophage inflammatory protein 1β
CXCL10: γ-Interferon Inducible Protein 10
IRF1: Interferon regulatory factor 1
IFNB1: Type I interferon-β1
ICAM-1: Intercellular adhesion molecule 1
STING: Stimulator of interferon response cGAMP interactor
AMD: Age-related macular degeneration
DAMPs: Damage-associated molecular patterns
RPE: Retinal pigment epithelium
dsDNA: Double-stranded DNA
ER: Endoplasmic-reticulum

## References

[1] Klaassen I, Van Noorden CJF, Schlingemann RO (2013). Molecular basis of the inner blood-retinal barrier and its breakdown in diabetic macular edema and other pathological conditions. Prog Retin Eye Res.

[2] Smith JR, David LL, Appukuttan B, Wilmarth PA (2018). Angiogenic and immunologic proteins identified by deep proteomic profiling of human retinal and choroidal vascular endothelial cells. Potential targets for new biologic drugs. Am J Ophthalmol.

[3] Tang J, Kern TS (2011). Inflammation in diabetic retinopathy. Prog Retin Eye Res.

[4] Kerur N, Fukuda S, Banerjee D, Kim Y, Fu D, Apicella I, Varshney A, Yasuma R, Fowler BJ, Baghdasaryan E, Marion KM, Huang X, Yasuma T, Hirano Y, Serbulea V, Ambati M, Ambati VL, Kajiwara Y, Ambati K, Hirahara S, Bastos-Carvalho A, Ogura Y, Terasaki H, Oshika T, Kim KB, Hinton DR, Leitinger N, Cambier JC, Buxbaum JD, Kenney MC, Jazwinski SM, Nagai H, Hara I, West AP, Fitzgerald KA, Sadda SR, Gelfand BD, Ambati J (2018). CGAS drives noncanonical-inflammasome activation in age-related macular degeneration. NAT MED.

[5] West AP, Khoury-Hanold W, Staron M, Tal MC, Pineda CM, Lang SM, Bestwick M, Duguay BA, Raimundo N, MacDuff DA, Kaech SM, Smiley JR, Means RE, Iwasaki A, Shadel GS (2015). Mitochondrial DNA stress primes the antiviral innate immune response. NATURE..

[6] West AP, Shadel GS, Ghosh S (2011). Mitochondria in innate immune responses. Nat Rev Immunol.

[7] Nakayama H, Otsu K (2018). Mitochondrial DNA as an inflammatory mediator in cardiovascular diseases. Biochem J.

[8] Rykova E, Sizikov A, Roggenbuck D, Antonenko O, Bryzgalov L, Morozkin E, Skvortsova K, Vlassov V, Laktionov P, Kozlov V (2017). Circulating DNA in rheumatoid arthritis. Pathological changes and association with clinically used serological markers. Arthritis Res Ther.

[9] Thurairajah K, Briggs GD, Balogh ZJ (2018). The source of cell-free mitochondrial DNA in trauma and potential therapeutic strategies. Eur J Trauma Emerg Surg.

[10] Zhang J, Liu Z, Liu J, Ren J, Sun T (2014). Mitochondrial DNA induces inflammation and increases TLR9/NF-κB expression in lung tissue. Int J Mol Med.

[11] West AP, Shadel GS (2017). Mitochondrial DNA in innate immune responses and inflammatory pathology. Nat Rev Immunol.

[12] Zhong Z, Liang S, Sanchez-Lopez E, He F, Shalapour S, Lin X, Wong J, Ding S, Seki E, Schnabl B, Hevener AL, Greenberg HB, Kisseleva T, Karin M (2018). New mitochondrial DNA synthesis enables NLRP3 inflammasome activation. Nature..

[13] Rodríguez-Nuevo A, Díaz-Ramos A, Noguera E, Díaz-Sáez F, Duran X, Muñoz JP, Romero M, Plana N, Sebastián D, Tezze C, Romanello V, Ribas F, Seco J, Planet E, Doctrow SR, González J, Borràs M, Liesa M, Palacín M, Vendrell J, Villarroya F, Sandri M, Shirihai O, Zorzano A (2018). Mitochondrial DNA and TLR9 drive muscle inflammation upon Opa1 deficiency. EMBO J.

[14] Bode C, Fox M, Tewary P, Steinhagen A, Ellerkmann RK, Klinman D, Baumgarten G, Hornung V, Steinhagen F (2016). Human plasmacytoid dentritic cells elicit a type I interferon response by sensing DNA via the cGAS-STING signaling pathway. Eur J Immunol.

[15] Riley JS, Quarato G, Cloix C, Lopez J, O'Prey J, Pearson M, Chapman J, Sesaki H, Carlin LM, Passos JF, Wheeler AP, Oberst A, Ryan KM, Tait SW (2018). Mitochondrial inner membrane permeabilisation enables mtDNA release during apoptosis. EMBO J.

[16] Mao Y, Luo W, Zhang L, Wu W, Yuan L, Xu H, Song J, Fujiwara K, Abe J, LeMaire SA, Wang XL, Shen YH (2017). STING-IRF3 triggers endothelial inflammation in response to free fatty acid-induced mitochondrial damage in diet-induced obesity. Arterioscler Thromb Vasc Biol.

[17] Long JZ, Svensson KJ, Bateman LA, Lin H, Kamenecka T, Lokurkar IA, Lou J, Rao RR, Chang MR, Jedrychowski MP, Paulo JA, Gygi SP, Griffin PR, Nomura DK, Spiegelman BM (2016). The secreted enzyme PM20D1 regulates lipidated amino acid uncouplers of mitochondria. Cell..

[18] Roubicek DA, Souza-Pinto NCD (2017). Mitochondria and mitochondrial DNA as relevant targets for environmental contaminants. Toxicology..

[19] Blajszczak C, Bonini MG (2017). Mitochondria targeting by environmental stressors: implications for redox cellular signaling. Toxicology..

[20] Roger AJ, Muñoz-Gómez SA, Kamikawa R (2017). The origin and diversification of mitochondria. Curr Biol.

[21] Youle RJ (2019). Mitochondria-Striking a balance between host and endosymbiont. Science (New York, N.Y.).

[22] Liu S, Feng M, Guan W (2016). Mitochondrial DNA sensing by STING signaling participates in inflammation, cancer and beyond. Int J Cancer.

[23] Nerlich A, Mieth M, Letsiou E, Fatykhova D, Zscheppang K, Imai-Matsushima A, Meyer TF, Paasch L, Mitchell TJ, Tönnies M, Bauer TT, Schneider P, Neudecker J, Rückert JC, Eggeling S, Schimek M, Witzenrath M, Suttorp N, Hippenstiel S, Hocke AC (2018). Pneumolysin induced mitochondrial dysfunction leads to release of mitochondrial DNA. Sci Rep-UK.

[24] Briston T, Roberts M, Lewis S, Powney B, Staddon JM, Szabadkai G, Duchen MR (2017). Mitochondrial permeability transition pore Sensitivity to opening and mechanistic dependence on substrate availability. Sci Rep-UK.

[25] Huang X, Zhai D, Huang Y (2001). Dependence of permeability transition pore opening and cytochrome C release from mitochondria on mitochondria energetic status. Mol Cell Biochem.

[26] Sun L, Wu J, Du F, Chen X, Chen ZJ (2013). Cyclic GMP-AMP synthase is a cytosolic DNA sensor that activates the type I interferon pathway. Science (New York, N.Y.).

[27] Xiao TS, Fitzgerald KA (2013). The cGAS-STING pathway for DNA sensing. Mol Cell.

[28] Chen M, Meng Q, Qin Y, Liang P, Tan P, He L, Zhou Y, Chen Y, Huang J, Wang R, Cui J (2016). TRIM14 inhibits cGAS degradation mediated by selective autophagy receptor p62 to promote innate immune responses. Mol Cell.

[29] Ishikawa H, Barber GN (2008). STING is an endoplasmic reticulum adaptor that facilitates innate immune signalling. Nature..

[30] Chen Q, Sun L, Chen ZJ (2016). Regulation and function of the cGAS-STING pathway of cytosolic DNA sensing. Nat Immunol.

[31] Ablasser A, Gulen MF (2016). The role of cGAS in innate immunity and beyond. J Mol Med (Berlin, Germany).

[32] Tanaka Y, Chen ZJ (2012). STING specifies IRF3 phosphorylation by TBK1 in the cytosolic DNA signaling pathway. Sci Signal.

[33] Fang R, Wang C, Jiang Q, Lv M, Gao P, Yu X, Mu P, Zhang R, Bi S, Feng J, Jiang Z (2017). NEMO-IKKβ are essential for IRF3 and NF-κB activation in the cGAS-STING pathway. J Immunol (Baltimore, Md. : 1950).

[34] Karthikkeyan G, Nareshkumar RN, Aberami S, Sulochana KN, Vedantham S, Coral K (2018). Hyperglycemia induced early growth response-1 regulates vascular dysfunction in human retinal endothelial cells. Microvasc Res.

[35] Maurer M, von Stebut E (2004). Macrophage inflammatory protein-1. Int J Biochem Cell Biol.

[36] Tatara Y, Ohishi M, Yamamoto K, Shiota A, Hayashi N, Iwamoto Y, Takeda M, Takagi T, Katsuya T, Ogihara T, Rakugi H (2009). Macrophage inflammatory protein-1beta induced cell adhesion with increased intracellular reactive oxygen species. J Mol Cell Cardiol.

[37] Antonelli A, Ferrari SM, Giuggioli D, Ferrannini E, Ferri C, Fallahi P (2014). Chemokine (C-X-C motif) ligand (CXCL)10 in autoimmune diseases. Autoimmun Rev.

[38] Takahashi S, Adachi K, Suzuki Y, Maeno A, Nakazawa M (2016). Profiles of inflammatory cytokines in the vitreous fluid from patients with rhegmatogenous retinal detachment and their correlations with clinical features. Biomed Res Int.

[39] Suzuki Y, Nakazawa M, Suzuki K, Yamazaki H, Miyagawa Y (2011). Expression profiles of cytokines and chemokines in vitreous fluid in diabetic retinopathy and central retinal vein occlusion. Jpn J Ophthalmol.

[40] Liu Y, Zeng L, Tian A, Bomkamp A, Rivera D, Gutman D, Barber GN, Olson JK, Smith JA (2012). Endoplasmic reticulum stress regulates the innate immunity critical transcription factor IRF3. J Immunol (Baltimore, Md. : 1950).

[41] Chen H, Sun H, You F, Sun W, Zhou X, Chen L, Yang J, Wang Y, Tang H, Guan Y, Xia W, Gu J, Ishikawa H, Gutman D, Barber G, Qin Z, Jiang Z (2011). Activation of STAT6 by STING is critical for antiviral innate immunity. CELL..

[42] Wang Y, Wang K, Yu S, Li Q, Li N, Lin P, Li M, Guo J (2015). Association of the TLR4 signaling pathway in the retina of streptozotocin-induced diabetic rats. Graefe's Arch Clin Exp Ophthalmol.

[43] Taniguchi T, Ogasawara K, Takaoka A, Tanaka N (2001). IRF family of transcription factors as regulators of host defense. Annu Rev Immunol.

[44] Amadi-Obi A, Yu C, Dambuza I, Kim S, Marrero B, Egwuagu CE (2012). Interleukin 27 induces the expression of complement factor H (CFH) in the retina. PLoS One.

[45] Eckhardt I, Weigert A, Fulda S (2014). Identification of IRF1 as critical dual regulator of Smac mimetic-induced apoptosis and inflammatory cytokine response. Cell Death Dis.

[46] Li T, Chen ZJ (2018). The cGAS-cGAMP-STING pathway connects DNA damage to inflammation, senescence, and cancer. J Exp Med.

