# Supplementary material for: Mitochondrial DNA drives noncanonical inflammation activation via cGAS–STING signaling pathway in retinal microvascular endothelial cells
Source: Cell Commun Signal. 2020 Oct 28;18:172. doi: 10.1186/s12964-020-00637-3 (PMC7592595; doi:10.1186/s12964-020-00637-3)
Supplement: Supplementary file 2 — Additional file 1: Table S1. Primer sequences used for real-time PCR in this study. (DOCX 15 kb) [file 12964_2020_637_MOESM2_ESM.docx]

Supplementary materials

Table S1 Primer sequences used for real-time PCR in this study.

| Gene | Forward（5‘→3’） | Reverse（3‘→5’） |
| --- | --- | --- |
| *cGAS* | GACTGGCTCGGCACAAAAGT | TAAGACGCCCACCTGTCTGG |
| *CCL4* | TTGTGATGGATTACTATGAGACCAG | ACATACTCATTGACCCAGGGC |
| *CXCL10* | CTGAGTGGGACTCAAGGGATC | TTCAGACACCTCTTCTCATTGTTC |
| *IRF1* | ATTAATTCCAACCAAATTCCAGG | TTGTATCGGCCTGTGTGAATG |
| *IFNB1* | GCCCTCTCCATCGACTACAAG | AAGACATTCTGGAGCATCACTTG |
| *ICAM-1* | GTCAAACGGGAGATGAATGGT | TCCTCTGGCGGTAATAGGTGT |
| *ACTIN* | CGTTGACATCCGTAAAGACCTC | TAGGAGCCAGGGCAGTAATCT |
| *GAPDH* | CGCTAACATCAAATGGGGTG | TTGCTGACAATCTTGAGGGAG |
