# Supplementary figures and images for: Mitochondrial DNA drives noncanonical inflammation activation via cGAS–STING signaling pathway in retinal microvascular endothelial cells
Source: Cell Commun Signal. 2020 Oct 28;18:172. doi: 10.1186/s12964-020-00637-3 (PMC7592595; doi:10.1186/s12964-020-00637-3)

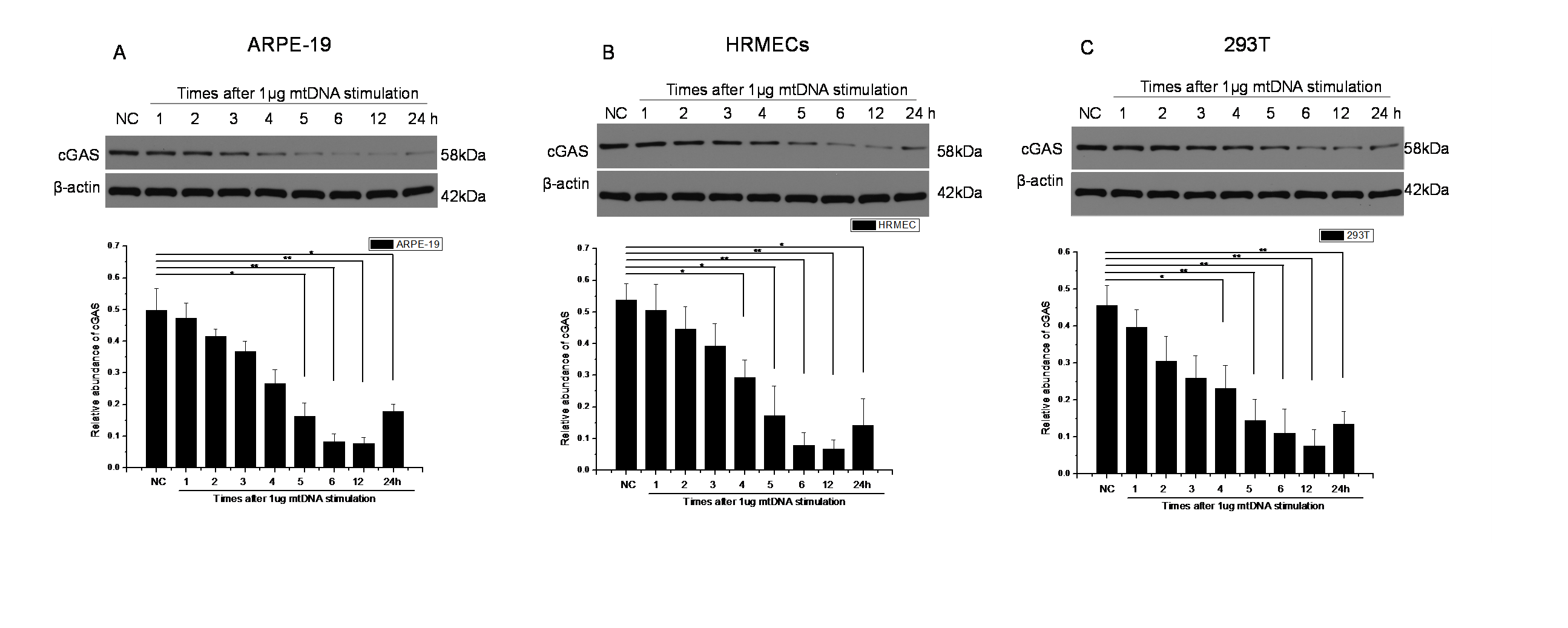

Supplement: Supplementary file 3 — Additional file 2. (TIFF 458 kb) [file 12964_2020_637_MOESM3_ESM.tiff]
